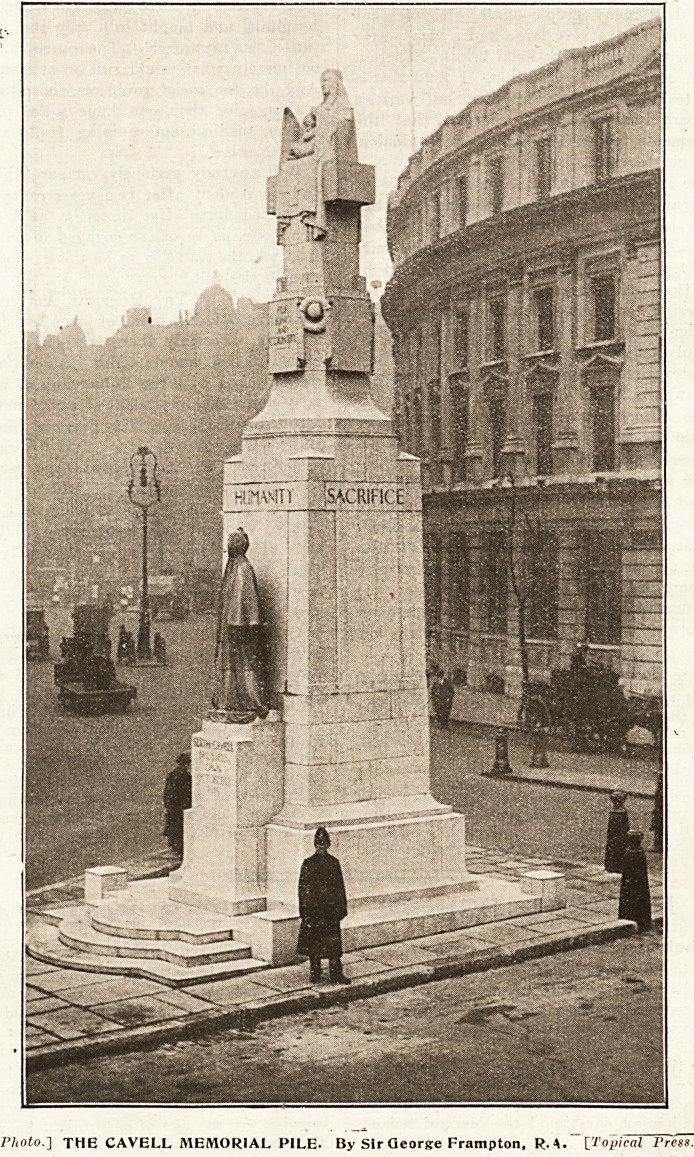# Unveiling the Edith Cavell Memorial

**Published:** 1920-03-20

**Authors:** 


### UNVEILING THE EDITH CAVELL MEMORIAL.

### A Beautiful and Memorable Ceremony.

ON March 17, St. Patrick's Day, a threatening morning relented, and warm, soft air replaced the bitter wind of recent days. It was a welcome relief to the large number of nurses in uniform, guests of honour, and others who gathered in St. Martin's Place long before the hour of noon to see the unveiling by Queen Alexandra of the statue by Sir George Frampton of Edith Cavell. A small daïs with red and gold canopy had been erected close to the great grey Cornish granite monument, against which the statue could be seen, covered by the Union Jack and the flag of Belgium. The English flag is Queen Alexandra's gift, and the other is one of two presented by Her Majesty the Queen of the Belgians. All three will find their ultimate home in the London Hospital, where Edith Cavell was trained. A smaller platform accommodated some special guests, and in other enclosures were some members of the Cavell family, deputations of nurses from various hospitals, a deputation from the Ecole Edith Cavell at Brussels, and one from Queen Alexandra's Hospital at Milbank of men sufficiently convalescent to stand through the ceremony.

*Photo.*] THE CAVELL MEMORIAL PILE. By Sir George Frampton, R.A. [*Topical Press.*

A guard of honour was, by permission of the King, furnished by the 2nd Battalion Coldstream Guards. The Matron, Mlle. de Meyer, and Nurse Lacomblé, headed the Belgian deputation. The Lord Mayor and Lady Mayoress were present to support the Mayor of Westminster, and amongst other well-known faces were those of the Rev. H. R. L. Sheppard, Vicar of St. Martin's Church, close by, Lady Burnham, and Mrs. Asquith. Princess Victoria accompanied Queen Alexandra, who arrived in an open carriage, and was evidently in excellent health. A table decorated with vases containing fine rose-red carnations and smilax had been placed beside a chair prepared for Her Majesty, but she preferred to remain standing throughout the eremony, which began by presentation of the members of the Memorial Committee.

These were :—The Lord Bishop of London; Sir George Frampton, Bart., R.A.; Sir Cyril Jackson, K.B.E.; Sir W. Watson Cheyne, K.C.P.; Sir George Welby, Bart., C.M.G.; Sir Frederick Taylor, Bart.; Mr. Norman Forbes Robertson; Sir John M. Le Sage; Mr. Fred. Miller; Alderman G. W. Tallents; Councillor G. Booth Hemming; Mr. John Hunt, Town Clerk of Westminster City Council; Mr. J. Hall Richardson, Hon. Sec.

Some members of the Cavell family were also presented, and the Belgian Ambassador presented General Doctor

(*Concluded on opposite page.*)

### Unveiling the Edith Cavell Memorial.

(*Continued from p. 589.*)

Maurice Funck, and the ladies of the deputation from the Cavell School at Brussels. Lord Burnham, as Chairman of the Memorial Committee, then read an address of welcome. After thanking Her Majesty for her presence and the gift of the flag, he explained that the memorial had been erected by subscriptions from readers of the *Daily Telegraph*. Many of these had been small in amount, coming from private soldiers who in some cases had themselves profited by Nurse Cavell's ministrations. Other gifts had come from overseas. We learn, in fact, that the sum asked for by the newspaper was generously and promptly over-subscribed—a fact which increased costs of labour and carriage made welcome. Lord Burnham went on to say that there had been some delay in completion, due to the difficulty found by Sir George Frampton in obtaining a sufficiently large block of white Carrara marble for the statue. He also recalled the fact that on the excellent site which the City of Westminster generously offered at once, there stood for some time, till its removal to Khartoum, the statue of another good servant of England overseas—General Gordon.

Queen Alexandra handed a written reply, after which the Bishop of London, who was robed and accompanied by his chaplain, dedicated the memorial in the Sacred Name, and offered a simple and beautiful prayer that the memory of Edith Cavell's unselfish labours in life, her steadfast faith, and her courage and fortitude in the hour of death might remain as an example to the people of her country, and lead them through life and death to rest in the Vision of God, "as our hope is that this our sister doth." A benediction followed, and a moment's silence, and then the Queen's hand drew away by a long cord the two flags which draped the figure. The hush that followed must have been to the sculptor worth more than much applause. The truth is that the dignified simplicity of the figure, tall in the white marble against the beautiful background which the grey of granite forms is eminently appealing.

The straight lines of the nurse's cloak, the hands simply dropped at *Attention*, the face with something of humour about the mouth in spite of its grave sweetness turned a little to the East combine to give the best possible realisation of the ideal representative of the great profession. There is no finer site in London on which the figure of a woman could stand, facing towards Westminster, where women have now to share the work of men in law-making, for betterment of social life in our own country, and her intervention in the world's affairs.

The granite shaft against which this figure stands has the shape of a cross roughly hewn, the arms rather hinted at than developed. On its summit a woman is seated nursing a child, emblematic not only of woman's care for childhood, but of England's for the smaller nations. *For King and Country* is inscribed beneath, and the word Humanity, one of the four words which appear on the four sides. The reverse bears the word Fortitude above a bas-relief of a lion trampling down the serpent, representing *Envy*, *Spite*, *Malice*, and *Treachery*. The emblem of St. Patrick, exterminator of serpents, was very suitably being sold beneath this face. The words Sacrifice and Devotion appear on the other faces, East and West.

After this unveiling, a military band played the melody of "Abide with Me," the hymn so intimately associated now with Nurse Cavell that it might well be taken as a Guild song by the nursing profession. A bugler played the "Last Post," a roll of drums and the Reveillé followed, and with thanks to Queen Alexandra the ceremony ended.

## Figures and Tables

**Figure f1:**